# An economy-ralated equity analysis of health service utilization by women in economically underdeveloped regions of western China

**DOI:** 10.1186/s12939-017-0667-y

**Published:** 2017-10-27

**Authors:** Yuyan Qian, Zhongliang Zhou, Ju’e Yan, Jianmin Gao, Yuping Wang, Xiaowei Yang, Yongjian Xu, Yanli Li

**Affiliations:** 1 School of Public Health, Health Science Center, Xi’an Jiaotong University, Xi’an, Shaanxi People’s Republic of China; 2 School of Humanities and Social Sciences, Xi’an Jiaotong University, Xi’an, Shaanxi People’s Republic of China; 3 School of Public Policy and Administration, Xi’an Jiaotong University, Xi’an, Shaanxi People’s Republic of China; 4 School of Law, Linyi University, Linyi, Shandong People’s Republic of China

**Keywords:** Health equity, women’s health services, Utilization, Chinese

## Abstract

**Background:**

The Chinese government has long been committed to eliminating the inequality in the utilization of health services; however, it still lacks an analysis or measurement of the economy-related inequality in the utilization of women’s health services.

**Methods:**

The economy-related utilization of health services in women aged 15 years and above was assessed by the horizontal inequity index of a two-week outpatient rate and annual inpatient rate from the 5th National Health Service Survey of Shaanxi Province. The concentration index of each factor was decomposed into the contribution of each factor to the economic-related inequality of health service utilization based on the Probit regression model.

**Results:**

The horizontal inequity indexes of the two-week outpatient rate was 0.0493, and the horizontal inequity indexes of the annual impatient rate was 0.0869. The contributions of economic status to the two indexes were 190.71% and 115.80%, respectively. Economic status, age, basic medical insurance, educational status, marital status, urban/rural area, and self-rated health were the main impact factors that affected the inequality in women’s health services utilization in Shaanxi.

**Conclusions:**

Health service utilization was different between women with different social demographic characteristics, and unequal health service utilization is evident among women in Shaanxi.

## Background

Women’s health is always a concern to the World Health Organization [1] due to some health challenges specific to women. For example, pregnancy is a risk factor for women’s health although normal pregnancy is not a disease. Also, inequality in education, employment, and income due to social discrimination against women results in an unequal access for women to get necessary health services and limits women’s ability to have better control of their health. This inequality may reduce the chance in women to acquire the best health care. In China, the health service survey in 1993, 1998, 2003, and 2008 showed that the two-week morbidity rate, outpatient rate, prevalence of chronic diseases, and inpatient rate were higher in females than males [2]. Therefore, females generally have a higher need and demand for health services than males.

Medical health care services are a major health production factor for consumers [3]. Increased availability of medical health care services is a key condition for improving the health of the population; additionally, advances in medical techniques can greatly enhance the overall life expectancy of a country. The inability to afford health care services for treatments of illness will not only result in the occurrence of health inequity, but may also lead individuals and families into a vicious cycle of poverty. Inequality and inequity widely exist in medical health care systems; this becomes far worse when combined with income inequality because the wealthy people can access more high-quality health care services, such as the expensive medications and examinations, than the poor people [4–7]. Many studies have reported unequal health service utilization between the poor people and the wealthy people [5–7], such as health service utilization being lower in low-income populations than in high-income populations [8–11], and high quality resource utilization being higher for the wealthy people than the poor people [12, 13]. Although economic status, health status, and health policy are all important factors that affect the utilization of health services [14–17], economic status contributes the most to the huge gap in health service utilization between poor and wealthy women [18]. Medicare insurance can improve the equity in the utilization of health services to some extent. Patients without health insurance are more likely to postpone their medical care and be denied of much needed medical care and medicines [19, 20]. In the past two decades, several medical insurance plans were developed by the government of China trying to cover the whole population. Since 2010, almost all the nation’s rural residents were covered under the New Rural Cooperative Medical Scheme, and this insurance has some effect on improving health service utilization in rural residents [21]. Age, occupation, and educational status are also important factors that affect health service utilization in women. Younger, well-educated, and higher income females have significantly higher health service utilization than older, less educated, and lower income females [22, 23].

The disparities in the utilization of health services result in inequality. Thus, differential needs must be taken into account for an inequality to be interpretable as an inequity. This means that the equity in utilization of health services ought to be allocated on the basis of health need, not socialeconomic characteristics such as income, ethnicity, etc. People with the same needs of health services should have the same chance to access health services; this is defined as “horizontal equity”. In contrast, people with different health service needs should have access to different health services; this is defiened as the “vertical equity”. In empirical studies, the measurement of vertical inequity of health services utilization remains underdeveloped [24], and health policies are also more likely to improve horizontal equity [25]. Therefore, well-developed measures should be used to empirically estimate the horizontal inequity of health services utilization in women.

Although the inequality of health service utilization was widely found in women of many countries and regions, most current studies focus on the equity of maternity health care utilization [26–29] among females of childbearing age. However, there is a lack of more comprehensive studies on health service utilization in the overall female population. This study was conducted to provide new insights into the formulation and adjustment of health policies in order to reduce economy-related inequality in women’s health service utilization and improve the female health equity.

## Methods

### Data source

The data in this study originated from the family health survey of the 5th National Health Service Survey (henceforth NHSS) of Shaanxi province. Shaanxi province is in the northwest region of China and covers an area of 205,800 km^2^; 48.36% of the 37.637 million population is female [30]. Shaanxi province is considered an economically underdeveloped region in China due to its low GDP per capita [31]. A multi-stage cluster random sampling method was used for the 5th NHSS in Shaanxi. At the first stage, 32 counties (districts) in the Shaanxi province were randomly selected. At the second stage, 160 townships were selected from the first stage samples. At the third stage, 320 villages (communities) were selected from the second stage samples. At the fourth stage, 20,700 families were chosen from the 320 villages (communities), and then 57,529 women aged 15 and older were finally selected for this study [32].

The survey was conducted by face-to-face interviews using the Family Health Questionnaire developed by the Health Statistics and Information Center of the Ministry of Health of China. The questionnaire covered general information about the socioeconomic and demographic characteristics of the family, demographic and insurance characteristics of the family members, self-reported diseases and injuries, and utilization of outpatient and inpatient health services. In order to ensure the quality of the survey, the National Health and Family Planning Commission of PRC organized experts to carry out the design and verify the investigation plan repeatedly. Each investigator was rigorously selected and trained in accordance with the requirements of the investigation program. The investigation program required the investigators to have a certain degree of professional knowledge, a high sense of responsibility, a serious mentality, a patient and meticulous attitude, and good social communication skills. After rigorously training, each investigator should understand the purpose, meaning, principles, and methods of the survey. In addition, the investigators should know the trouble-shoting that may arise during the investigation. A survey quality assessment team was established in each county (district) to randomly check the quality of the investigation. Survey supervisors re-interviewed 5% of the individuals to check the accuracy of the data. In this process, 14 key questions were asked again. The consistency rates of these key questions were over 95% between two interviews [33]. Finally, 8580 females from urban areas and 14,684 females from rural areas (23,264 females with an age 15 and older and correctly filled survey) were included in the data analysis.

### Indicators for measuring health services utilization

Based on a previous study [19] and our experience, the two-week outpatient rate and annual inpatient rate of women were selected for the indicators of health services utilization. In particular, the two-week outpatient rate = number of outpatients within two weeks / total surveyed individuals * 100% (formula 1).

Annaul inpatient rate = number of annual inpatients / total surveyed individuals * 100% (formula 2).

### Equity analysis of health services utilization in women

#### Concentration index

The concentration index is the most scientific and effective method for measuring health equity [25]. In this study, the concentration index was adopted to analyze and compare the utilization of health services between women with different economic status in order to determine whether their health services utilization was equal. Concentration index (C) was calculated as:*C* = 2 cov(*x*, *h*)/*μ* (formula 3). Where *x* is the ranking of economic status, *h* is the ranking of health service utilization, and *μ* is the mean of health servie utilization.

#### Decomposition of concentration index

The concentration index of each factor was decomposed into the contribution of each factor to the inequality in health service utilization. This method has been widely used in the analysis of impact factors for health equity, and has shown a relatively accurate reflection of the inequality in the distribution of health service utilization under different socioeconomic conditions [32, 34]. Based on the Probit regression model, the concentration index of each factor was decomposed into the contribution of each factor to the inequality in health service utilization. When a variable is the only impact factor, a positive contribution rate indicates that the variable has increased the inequality in the results and vice versa. The two-week outpatient rate and annual inpatient rate were set as the dependent variables, and concentration indexes of health service utilization were decomposed by using the Probit regression model. The formula of the model was:

4 $$ {y}_i={a}^m+\sum \limits_j{\beta}_j^m{x}_{ji}+\sum \limits_k{\gamma}_k^m{z}_{ki}+{\mu}_i $$

The formula for the decomposition of concentration index:

5 $$ C=\sum \limits_j\left({\beta}_j^m{\overline{x}}_j/\mu \right){C}_j+\sum \limits_k\left({\gamma}_k^m{\overline{z}}_k/\mu \right){C}_k+{GC}_{\mu }/\mu $$

where *C* is the non-standardized concentration index of *y*, *C*_*j*_ is the concentration index of *x*_*j*_Cjxjγk, *C*_*k*_is the concentration index of *γ*_*k*_ γk, *GC*_*μ*_ is the concentration index of the residual term, and ${\overline{x}}_j$ and ${\overline{z}}_k$ are the means of *x*_*j*_ and *z*_*k*_, respectively.

#### Horizontal inequity index

The concentration index (*C*) of health service utilization was equal to the weighted sum of the concentration index of the “need” variable and the “control” variable (the residual term was not considered). The “need” variable included the age and sickness in two weeks, chronic diseases, and self-rated health. The “control” variable included the educational status, marital status, employment status, economic status, basic medical insurance, urban/rural areas, regions, and the adjusted number of family members (Table 1). The product of each “need” and “control” variable’s concentration index and its weighting indicated the contributions of these variables to the inequality in health service utilization. The horizontal inequity index was calculated by subtracting the contribution of the “need” variable from the concentration index of health service utilization [35].

**Table 1:** Description of variables (N/% or means)

		N	percent(%)/mean
Healthcare Utilization			
Two-week outpatient rate	Visit	2300	9.89
Annual inpatient rate	Hospitalized	2623	11.27
Need variables			
Age(years)	15–24*	2456	10.56
	25–34	2967	12.75
	35–44	4610	19.82
	45–54	5215	22.42
	55–64	4233	18.20
	65-max	3783	16.26
Illness in last two weeks	Not sick*	18,052	77.60
	Sick	5212	22.40
Chronic disease	Not sick*	17,432	74.93
	Sick	5832	25.07
Self-rated health	Good*	16,382	70.42
	Bad	6882	29.58
Control variables			
Educational status	Illiterate*	5038	21.66
	Primary school	5750	24.72
	Junior high school	8010	34.43
	Senior high school	3012	12.95
	Junior college and above	1454	6.25
Marital status	Unmarried*	2219	9.54
	married	18,680	80.30
	Widowed and divorced	2365	10.17
Employment status	Employed*	16,179	69.55
	retirement	1385	5.95
	student	1053	4.53
	unemployed	4647	19.98
Economic status	Lowest group*	4653	20.00
	Lower group	4654	20.01
	Medium group	4792	20.60
	Higher group	4536	19.50
	Highest group	4629	19.90
Basic medical insurance	UEBMI	2035	8.75
	URBMI	1341	5.76
	NRCMS	18,210	78.28
	URRBMI	1293	5.56
	Uninsured	385	1.65
Urban/rural area	rural*	14,684	63.12
	urban	8580	36.88
region	Shannan*	7314	31.44
	Guanzhong	12,187	52.39
	Shanbei	3763	16.18
Adjusted family size		23,264	2.20

*Reference levels in the Probit regression. UEBMI: Urban Employee Basic Medical Insurance; URBMI: Urban Residents Basic Medical Insurance; NRCMS: New Rural Cooperative Medical Scheme; URRBMI: Urban and Rural Residents Basic Medical Insurance

### Statistical anylysis

The difference in health service utilization between women with different social demographics was analyzed using the *χ* ^*2*^ test. A *p* < 0.05 was considered statistical significance.

## Result

### General data on health service utilization by women in Shaanxi province

The two-week outpatient rate and annual inpatient rate in women aged 15 and older in Shaanxi were 9.89% and 11.27%, respectively. Among women with different ages, the two-week outpatient rate increased as age increased. The annual inpatient rates in the 25–34 years, 55–64 years, and 65 years old and above group were higher than that in the 15–24 years, 35–44 years, and 45–54 years old group. Among women with different educational statuses, the two-week outpatient rate and annual inpatient rate were higher in the illiterate group than that in other groups; both rates also showed a trend to reduce with an increase in educational level. In addition, the two-week outpatient rate and annual inpatient rate were higher in widowed and divorced women than in women with other marital statuses. The two-week outpatient rate was highest in unemployed women, while the annual inpatient rate was highest in retired women. The two-week outpatient rate and the annual inpatient rate were increased with an increase in economic level, but there were no statistically significant differences (*p* > 0.05) in two-week outpatient rates between women with different economic status. Among women with different basic medical insurance, the two-week outpatient rate was highest in women with the new rural cooperative medical insurance, and the annual inpatient rate was highest in women with the urban employee medical insurance. The annual inpatient rate was higher in women living in rural areas than in women living in urban areas, but there was no statistically significant difference in the two-week outpatient rate between the two areas (*p* > 0.05). The two-week outpatient rate was the highest in women living in the southern region of Shaanxi, while annual inpatient rate was the highest in women living in the northern region of Shaanxi. Annual inpatient rate of women with illness in two weeks was higher than in women without illness. Finally, the two-week outpatient rate and the annual inpatient rate were both higher in women with chronic diseases and poor self-rated health than those without chronic diseases and with good self-rated health, respectively (See Table 2).

**Table 2:** Utilization of health services

	Two-week outpatient rate (%)	*χ* ^2^	*p*	Annual inpatient rate (%)	*χ* ^2^	*p*
Age group		521.243	<0.001		359.944	<0.001
15–24	2.85			8.06		
25–34	3.77			13.38		
35–44	7.94			6.79		
45–54	10.66			8.94		
55–64	13.96			13.18		
65-max	15.99			18.27		
Educational status		307.398	<0.001		109.540	<0.001
Illiterate	14.65			14.63		
Primary school	12.10			12.21		
Middle school	7.87			10.05		
High school	5.94			8.13		
Junior college and above	3.92			9.22		
Marital status		275.300	<0.001		244.843	<0.001
Unmarried	3.24			2.00		
married	9.68			11.77		
Widowed and divorced	17.76			15.98		
Employment status		126.767	<0.001		262.982	<0.001
employed	9.24			10.16		
retirement	12.06			17.83		
student	2.85			1.00		
unemployed	13.08			15.45		
Economic status		9.330	0.053		60.457	<0.001
Minimum group	9.11			10.02		
Lower group	9.39			10.85		
Medium group	9.81			10.12		
Higher group	10.52			12.37		
Highest group	10.63			14.32		
Basic medical insurance		25.017	<0.001		11.049	0.026
UEBMI	8.01			12.78		
URBMI	9.32			11.93		
NRCMS	10.36			11.22		
URRMI	7.97			9.74		
Uninsured	5.97			8.57		
Urban and rural		0.422	0.516		10.275	0.001
urban	9.98			10.77		
rural	9.72			12.14		
Region		59.502	<0.001		19.177	<0.001
Shannan	11.72			11.4		
Guanzhong	9.63			10.61		
Shanbei	7.18			13.18		
Illness in last two weeks					748.640	<0.001
Not sick				8.23		
Sick				21.83		
Chronic disease		2000.000	<0.001		938.262	<0.001
Not sick	4.83			7.60		
Sick	25.00			22.26		
Self-rated health		816.153	<0.001		555.746	<0.001
Good	6.26			8.11		
Bad	18.51			18.82		

### Inequity in health service utilization by women

Table 3 shows the concentration indices of the health service utilization indicators. The concentration index of both the two-week outpatient rate (0.0366) and the annual inpatient rate (0.0772) was positive, indicating a pro-rich inequality in the utilization of health services in women. Decomposition of the concentration index of each health service utilization indicator is shown in Table 4. The contribution rate is the percentage of each variable’s contribution to the inequality in the two-week outpatient rate and the annual inpatient rate. Economic status showed the greatest contribution rate toward the inequality in the two indicators (190.71% and 115.80%, respectively) and the contribution rate was increased with an increase in economic level. The contribution rate of basic medical insurance was found to be the second largest contribution rate to the inequality in two-week outpatient rate and annual inpatient rate (−53.28% and −23.23%, respectively). The contribution rate of educational status, urban/rural areas, and self-rated health to the two-week outpatient rate were −30.60%, 24.04%, and −30.60%, respectively. The contribution rate of marital status to the annual inpatient rate was −21.24%. The absolute values of the contribution rate of the other factors were less than 20%.

**Table 3:** Concentrate index (CI) of health service utilization

	CI	SE.	95% CI	
Two-week outpatient rate	0.0366	0.0114	0.0142	0.0591
Annual inpatient rate	0.0772	0.0106	0.0565	0.0980

**Table 4:** Concentration index decomposition of utilization of health physical examination

	Two-week outpatient rate				Annual inpatient rate			
	dF/dx	CI	Contribution	%	dF/dx	CI	Contribution	%
Age (years) (Reference level = 15–24)				8.20				2.33
25–34	0.000	0.0899	0.0000	0.00	−0.031***	0.0899	−0.0063	−8.16
35–44	0.008*	0.1228	0.0061	16.67	−0.052***	0.1228	−0.0330	−42.75
45–54	0.006	0.0297	0.0015	4.10	−0.039***	0.0297	−0.0091	−11.79
55–64	0.004	0.1136	−0.0038	−10.38	−0.029***	0.1136	0.0259	33.55
65-max	0.001	0.1308	−0.0008	−2.19	−0.022***	0.1308	0.0243	31.48
Education status (Reference level = Illiterate)				−30.60				−2.85
Primary school	−0.001	0.0873	0.0002	0.55	−0.002	0.0873	0.0004	0.52
Middle school	−0.008	0.0370	−0.0011	−3.01	−0.010	0.0370	−0.0011	−1.42
High school	−0.020**	0.2121	−0.0055	−15.03	−0.017*	0.2121	−0.0040	−5.18
Junior college and above	−0.018	0.4313	−0.0048	−13.11	0.011	0.4313	0.0025	3.24
Marital status (Reference level = Unmarried)				−7.65				−21.24
Married	0.017	0.0138	0.0019	5.19	0.118***	0.0138	0.0114	14.77
Widowed and divorced	0.039**	0.1189	−0.0047	−12.84	0.264***	0.1189	−0.0278	−36.01
Employment status (Reference lvel = Employed)				7.10				8.81
Retirement	−0.002	0.4140	−0.0006	−1.64	0.040***	0.4140	0.0085	11.01
Student	0.013	0.0827	0.0005	1.37	−0.052***	0.0827	−0.0017	−2.20
Unemployed	−0.001	0.0934	0.0027	7.38	0.013*	0.0934	−0.0020	−2.59
Economic status (Reference level = Minimum group)				190.71				115.80
Lower group	0.009	0.3998	−0.0073	−19.95	0.019**	0.3998	−0.0134	−17.36
Medium group	0.017**	0.0061	0.0002	0.55	0.013*	0.0061	0.0001	0.13
Higher group	0.030***	0.4071	0.0237	64.75	0.039***	0.4071	0.0269	34.84
Highest group	0.033***	0.8011	0.0532	145.36	0.055***	0.8011	0.0758	98.19
Basic medical insurance (Reference level = UEBMI)				−53.28				−23.32
URBMI	0.014	0.2865	0.0023	6.28	0.016	0.2865	0.0023	2.98
NRCMS	0.033***	0.0576	−0.0149	−40.71	0.029**	0.0576	−0.0115	−14.90
URRMI	0.038*	0.3651	−0.0077	−21.04	0.050**	0.3651	−0.0089	−11.53
Uninsured	0.020	0.2275	0.0008	2.19	0.004	0.2275	0.0001	0.13
Urban and rural (Reference level = Rural)				24.04				4.53
Urban	0.012**	0.2032	0.0088	24.04	0.005	0.2032	0.0035	4.53
Region (Reference level = Shannan)				−15.85				3.24
Guanzhong	−0.005	0.0281	0.0007	1.91	0.000	0.0281	0.0000	0.00
Shanbei	−0.028	0.1405	−0.0065	−17.76	0.012*	0.1405	0.0025	3.24
Adjusted family size	−0.007**	0.0194	0.0031	8.47	−0.002	0.0194	0.0008	1.04
Illness in last two weeks (Reference level = Not sick)								2.59
Sick		0.0245			0.041***	0.0245	0.0020	2.59
Chronic disease (Reference level = Not sick				−12.30				−2.85
Sick	0.156***	0.0115	−0.0045	−12.30	0.090***	0.0115	−0.0022	−2.85
Self-rated health (Reference level = Good)				−30.60				−14.64
Bad	0.053***	0.0709	−0.0112	−30.60	0.062***	0.0709	−0.0113	−14.64

*P < 0.05, **P < 0.01, ***P < 0.001

**Fig. 1:**
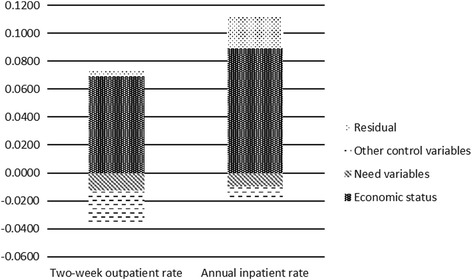
The contributions of economic status and residual term on the two-week outpatient rate and the annual inpatient rate

As shown in Fig. 1, the contributions of economic status and residual term on the two-week outpatient rate and the annual inpatient rate were located above the level of the horizontal fair line, indicating that the contribution of this variable made the concentrate index of two-week outpatient rate and the annual inpatient rate more conducive to the wealthy people. The other control and need variables were below the horizontal fair line, indicating that their contribution made the concentration index for two-week outpatient rate and annual inpatient rate more favorable for the poor people.

After controlling the effect of the “need” variables on the inequality of health service utilization, the horizontal inequity index of the two-week outpatient rate and annual inpatient rate were 0.0493 and 0.0869, respectively, indicating the presence of a pro-rich inequity among health service utilization (Table 5). Furthermore, the horizontal inequity was higher in the annual inpatient rate than in the two-week outpatient rate.

**Table 5:** Horizontal inequity of health service utilization

	Two-week outpatient rate	Percentage (%)	Annual inpatient rate	Percentage (%)
Need variables	−0.0127	−34.70	−0.0097	−12.56
Control variables	0.0450	122.95	0.0644	83.42
Residual	0.0043	11.75	0.0225	29.15
Total	0.0366	100.00	0.0772	100.00
Horizontal inequity	0.0493		0.0869	

## Disscussion

This study found that health service utilization was increased in women as age increased, which was consistent with the findings from a previous study [36]. In particular, the annual inpatient rate was highest in women with an age 25–34 year old due to the high rate of pregnancy. The two-week outpatient rate and annual inpatient rate increased, but educational level decreased due to the high incidence of common gynecological diseases among poorly educated women living in rural areas [37], and the high awareness of health care among highly educated women. Given that divorce and widowing have negative effects on women’s health [38], both the two-week outpatient rate and the annual inpatient rate were higher in divorced and widowed women than marital women in our study. It is commonly accepted that unemployment has a negative effect on health [39]. This study showed that health service utilization was significantly higher for the unemployed and retired woman. The annual inpatient rate was increased, specifically in the economic status of women, because women’s financial capability affects the inpatient services. While the new rural cooperative medical plan has improved the utilization of outpatient services, the urban employee medical insurance has increased the utilization of inpatient services in women due to a high percentage of reimbursement [40]. Sickness within two weeks, suffering from chronic diseases, and poor self-rated health all significantly increased the utilization of health services. To the best of our knowledge, this is the first study to examine the economy-related equity in health service utilization in women using a large-scale representative sample.

In the current study, the concentration index of both the two-week outpatient rate and the annual inpatient rate were positive values, suggesting that the indicators of health service utilization in women aged 15 and above were concentrated in the rich women in Shaanxi. In other words, women with higher economic status can utilize more health services than women with lower economic status, demonstrating a pro-rich inequality in health service utilization. Moreover, the concentration index of the annual inpatient rate was higher than that of the two-week outpatient rate, suggesting a much greater inequality in inpatient service utilization. Economic status has the greatest contribution to inequality in the two-week outpatient rate and the annual inpatient rate. Consistent with previous reports [41], elevated economic levels increased the pro-rich inequality in health service utilization, and the gap between the rich and poor people is still the main impact factor of inequality in health service utilization. This was demonstrated that the wealthy people has greater economic advantages in health service utilization than the poor people [42].

Some studies suggest that the current insurance systems are filled with problems such as supplier-induced demand and inability to effectively reduce the risk of self-financed expenditure or catastrophic expenditure [43], as well as having a limited effect on reducing the inequality in health service utilization [44]. In this study, the Urban and Rural Residents Basic Medical Insurance is an insurance system comprised of Urban Residents Basic Medical Insurance (mainly for minors and unemployed individuals who were not covered under the Urban Employee Medical Insurance) and the New Rural Cooperative Medical Scheme. However, given that the insured subjects were mostly low-income individuals, these two insurances have somewhat stimulated the demand of health services in low-income women so that the utilization of both outpatient and inpatient services were increased [45]. This study indicated that the New Rural Cooperative Medical Scheme and the Urban and Rural Residents Basic Medical Insurance can reduce the wealth-biased inequality in health service utilization to some extent, and basic medical insurances are still an important health policy for reducing the inequality in health service utilization [36, 46].

Our findings indicated that educational status and poor self-rated health reduced the pro-rich inequality in the two-week outpatient rate, while urban residents increased the pro-rich inequality in the two-week outpatient rate. In today’s society, an individual’s economic strength includes not only the possession of material capitals, but also the possession of highly intelligent and highly skilled human capital. An important condition for acquiring such capital is the individual’s educational level. The educational level is not only the basis for measuring a woman’s development and social participation, but it is also an important indicator of a woman’s social and economic status [47]. Women with lower educational level have relatively lower economic status, and are more vulnerable to diseases [48]. Therefore, the two-week outpatient rate was mostly concentrated among poorly educated and low-income women, and the increase in educational level conversely reduced the pro-rich inequality in the two-week outpatient rate. Previous studies demonstrated that low-income individuals have relatively poor self-rated health [49], and women with poor self-rated health have higher outpatient service utilization than those with good self-rated health [36]. Thus, it is not surprising that the poor self-rated health reduced the pro-rich inequality in the two-week outpatient rate. Because the per capita income of urban population was higher than that of the rural population [50], women living in urban areas have a greater financial advantage in outpatient service utilization than those living in rural areas. Therefore, the urban residents increased the pro-rich inequality in the two-week outpatient rate.

This study revealed that marital status reduced the pro-rich inequality of inpatient service utilization. Compared to married women, divorced and widowed women have lower income, and given the need to take care of the family, many widowed or divorced women succumb to low-income as a result of the inability or unwillingness to take up a higher income full-time job [51]. Since widowed and divorced women have poorer health which increased their health service utilization, and therefore reduced the wealth-favored inequality in inpatient service utilization.

Differences in socioeconomic status have been widely known to cause differences in health service utilization, but they may not fully reflect the inequality in health service utilization. In order to accurately determine the socioeconomic status-related inequality in health service utilization, horizontal inequity should be measured. It means that the health service needs should be normalized in the surveyed individuals who have the same health service needs, but with different socioeconomic status. In this study, after eliminating the effect of the “need” variables, the horizontal inequity index showed that the pro-rich inequality in health service utilization not only still existed among women with the same health service needs, but also this inequality was even higher than that before subtracting the effect of the “need” variables. The wealthy people have greater health service utilization than poor people, and the horizontal inequity of the annual inpatient rate was higher than that of the two-week outpatient rate.

We acknowledge that the current study has a few limitations. First, the data were only originated from the Shaanxi province and may be biased due to regional differences. Thus, the conclusions drawn in this study may not be applicable to the entire nation. Second, the data on health service utilization and household consumption expenditure were all self-reported, which may be prone to memory biases. Nonetheless, self-reported health service utilization and household consumption expenditure have been widely adopted in large-scale family surveys [7, 11, 19, 51, 52]. Finally, due to the availability of data, the present study did not consider all factors that may influence health service utilization, such as the nature of the occupation, individual’s awareness of disease, and attitudes towards medical institutions, and so on. Omitting these factors may lead to biases in estimating the equity of health service utilization.

## Conclusions

Pro-rich inequality in health service utilization is still found among women in Shaanxi province, and economic status is the central factor of the inequality. Thus, the government should politically narrow the gap in the per capita economic status of women, protect the low-income women from discrimination in the labor market, and improve the basic medical insurance and social security systems.

## References

[1] Women today’s evidence tomorrow’s agenda. World Health Organization. 2009. http://www.who.int/gender-equity-rights/knowledge/9789241563857/en/. Accessed 15 Mar 2017.

[2] An analysis report of National Health Services Survey in China. Center for Health Statistics and Information. 2008. http://www.moh.gov.cn/mohwsbwstjxxzx/s8211/201009/49165.shtml. Accessed 15 Mar 2017.

[3] Wang J, Wang XW (2008). Health capital new development of human capital. Chinese Health Economics.

[4] Gwatkin DR, Bhuiya A, Victora CG (2004). Making health systems more equitable. Lancet.

[5] Zere E, Moeti M, Kirigia J, Mwase T, Kataika E (2007). Equity in health and healthcare in Malawi: analysis of trends. BMC Public Health.

[6] Ghosh S (2014). Equity in the utilzation of healthcare services in India: evidence from National Sample Survey. Int J Health Policy Manag.

[7] Zhou Z, Su Y, Gao J, Campbell B, Zhu Z, Xu L (2013). Assessing equity of healthcare utilization in rural China: results from nationally representative surveys from 1993 to 2008. Int J Equity Health.

[8] Asada y, Kephart G (2007). Equity in health service use and intensity of use in Canada. BMC Health Serv Res.

[9] Park JM (2016). Equity in the utilization of physician and inpatient hospital services: evidence from Korean health panel survey. Int J Equity Health.

[10] Gao J, Tang S, Tolhurst R, Rao K (2001). Changing access to health services in urban China: implications for equity. Health Policy Plan.

[11] Zhou Z, Gao J, Fox A, Rao K, Xu K, Xu L (2011). Measuring the equity of inpatient utilization in Chinese rural areas. BMC Health Serv Res.

[12] Flatø H, Zhang H (2016). Inequity in level of healthcare utilization before and after universal health coverage reforms in China: evidence from household surveys in Sichuan Province. Int J Equity Health.

[13] Hassanzadeh J, Mohammadbeigi A, Eshrati B, Rezaianzadeh A, Rajaeefard A (2013). Determinants of inequity in health care services utilization in Markazi Province of Iran. Iran Red Crescent Med J.

[14] Macinko J, Lima-Costa MF (2012). Horizontal equity in health care utilization in Brazil, 1998-2008. Int J Equity Health.

[15] Wu J, Liu J, Zhu B, Mao Y (2015). Assessing equity of health service utilization of rural residents in China: a case study of z county. Shaaxi Province Value in health.

[16] Park JM (2014). Chronic diseases, health status and health service utilization among Koreans. Health.

[17] Mohammadbeigi A, Hassanzadeh J, Eshrati B, Rezaianzadeh A (2013). Decomposition of inequity determinants of healthcare utilization. Iran Public Health.

[18] Kim C, KMA S, Salehi AS, Zeng W (2016). An equity analysis of utilization of health services in Afghanistan using a national household survey. BMC Public Health.

[19] Tikkanen RS, Woolhandler S, Himmelstein DU, Kressin NR, Hanchate A, Lin MY, McCormick D, Lasser KE. Hospital Payer and Racial/Ethnic Mix at Private Academic Medical Centers in Boston and New York City International Journal of Health Services. 2017; 10.1177/0020731416689549. 10.1177/0020731416689549 PMC6090544 28152644

[20] Zhou Z, Zhu L, Zhou Z, Li Z, Gao J, Chen G (2014). The effects of China’s urban basic medical insurance schemes on the equity of health service utilization: evidence from Shaanxi province. Int J Equity Health.

[21] Qian J, Gao J, Rao K, Wagstaff A, Lindelow M (2008). Study on the impact of new rural cooperative medical system on farmers' health service utilization. Chinese Journal of Health Statistics.

[22] Jiang X (2014). Gradually eliminate differences to improve the health status of Chinese women. Chinese Women’s Movement.

[23] Jiang X (2014). Gender differences in the health level of Chinese citizens - an analysis based on the third survey data of Chinese women's social status. Journal of China Women’s University.

[24] Sutton M (2002). Vertical and horizontal aspects of socio-economic inequity in general practitioner contacts in Scotland. Health Econ.

[25] Wagstaff A, Doorslaer EV (2000). Measuring and testing for inequity in the delivery of health care. J Hum Resour.

[26] Houweling TA, Ronsmans C, Campbell OM, Kunsta AE (2007). Huge poor- rich inequalities in maternity care: an international comparative study of maternity and child care in developing countries. Bull World Health Organ.

[27] Anwar I, Sami M, Akhtar N, Chowdhury ME, Salma U, Rahman M, Koblinsky M (2008). Inequity in maternal health-care services: evidence from home-based skilled-birth-attendant programs in Bangladesh. Bull World Health Organ.

[28] Han J, Shi S, Liu X, Zhang J, Su C, Ye M (2001). An investigation on the utilization of health service for married women in Wuhan. Chinese Health Service Management.

[29] Wu J, Ji L, Ren A, Zheng J, Chen X, Li Z (2003). Equity in perinatal health cares in 21 Chinese southern countries. Chinese Journal of Reproductive Health.

[30] China Statistical Yearbook. National Bureau of Statistics of the People’s Republic of China. 2014. http://www.stats.gov.cn/tjsj/ndsj/2014/indexch.htm. Accessed 15 Mar 2017.

[31] Xu Y, Gao J, Zhou Z, Xue Q, Yang J, Luo H (2016). Measurement and explanation of socioeconomic inequality in catastrophic health care expenditure: evidence from the rural areas of Shaanxi Province. BMC Health Serv Res.

[32] Zhou Z, Fang Y, Zhou Z, Li D, Wang D, Li Y, et al. Assessing income-related health inequality and horizon inequity in China. Social Indicators Research. 2016; doi:10.1007/s11205-015-1221-1.

[33] Xu Y, Gao J, Zhou Z, Xue Q, Yang J, Luo H, Li Y, Lai S, Chen G (2015). Measurement and explanation of socioeconomic inequality in catastrophic health care expenditure: evidence from the rural areas of Shaanxi Province. BMC Health Serv Res.

[34] Asada Y (2005). Assessment of the health of Americans: the average health-related quality of life and its inequality across individuals and groups. Popul Health Metrics.

[35] Zhou Z, Gao J, Xue Q, Yang XW, Yan J (2009). Effects of rural mutual health care on outpatient service utilization in Chinese village medical institutions: evidence from panel data. Health Econ.

[36] Rattay P, Butschalowsky H, Rommel A, Prütz F, Jordan S, Nowossadeck E (2013). Utilisation of outpatient and inpatient health services in Germany. Bundesgesundheitsbl.

[37] Li T, Chen R (2005). Overview of clinical epidemiology of cervical cancer. Practical Journal of Clinical Medicine.

[38] Zhang G (2006). The impact of changes in marital status on women's health. Chin J Public Health.

[39] Gao M (2004). Econometric analysis on how unemployment affect health. Chinese Primary Health Care.

[40] Gao J, Chen X, Pei Y, Yan J, Wang M (2011). Comparative analysis on the residents’ health service need and utilization under three basic medical insurance system. Chinese Journal of Health Policy.

[41] Hansen AH, Halvorsen PA, Ringberg U, Forde OH (2012). Socio-economic inequalities in health care utilisation in Norway: a population based cross-sectional survey. BMC Health Serv Res.

[42] Tang J, Zhang Y, Guo Z (2005). Analyzing the equity of medical service use in Zhejiang province. Health Economics Research.

[43] Wagstaff A, Lindelow M, Gao J, Xu L, Qian J (2007). Extending health insurance to the rural population: an impact evaluation of China’s new cooperative medical scheme. J Health Econ.

[44] Han B, Yuan Z, Liao X, Xiao Y, Hong Y (2010). Follow-up survey on the influence of the new rural cooperative medical system to the equity of health service utilization. Chinese Journal of Social Medicine.

[45] Su G, Kang L, Wu X (2008). Analysis on the health needs of menopausal women in rural areas after the implementation of NRCMS in Beidaihe district. Maternal and Child Health Care of China.

[46] Savitha S (2014). Effect of micro health insurance on access and utilization of health Services in Karnataka. Open Medicine Journal.

[47] Ma W (2005). The history, current situation and problems of the development of Chinese women's higher education. Research on Educational Development.

[48] Williams LK, Andrianopoulos N, Cleland V, Crawford D, Ball K (2013). Associations between education and personal income with body mass index among Australian women residing in disadvantaged neighborhoods. Am J Health Promot.

[49] Lu Y, Zhao H (2009). Study on self-rated health of people with different income in Sunan area of Jiangsu. Chinese Public Health.

[50] Statistical Communique on National Economic and Social Development. National Bureau of Statistics of the People’s Republic of China. 2014. http://www.stats.gov.cn/tjsj/zxfb/201402/t20140224_514970.html. Accessed 15 Mar 2017.

[51] Shirahase S, Raymo JM (2014). Single mothers and poverty in Japan: the role of intergenerational Coresidence. Social Forces.

[52] Mohammadbeigi A., Hassanzadeh J., Eshrati B., Rezaianzadeh A. Decomposition of inequity determinants of healthcare utilization, Iran. Public Health. 2013: doi:10.1016/j.puhe.2013.01.001. 10.1016/j.puhe.2013.01.001 23608021

